# Attitudes Towards Standardization of Mesenchymal Stromal Cells—A Qualitative Exploration of Expert Views

**DOI:** 10.1093/stcltm/szad056

**Published:** 2023-09-15

**Authors:** Alison J Wilson, Nik Brown, Emma Rand, Paul G Genever

**Affiliations:** Department of Biology, University of York, York, UK; Department of Sociology, University of York, York, UK; Department of Biology, University of York, York, UK; Department of Biology, University of York, York, UK

**Keywords:** ATMP, mesenchymal stromal cell, standard, standardization, cell therapy, translation

## Abstract

Pharmacopoeial standards ensure quality control of established medicines. It is widely believed that translation of cell therapy medicines will be facilitated by defining and adopting relevant standards. Mesenchymal stromal cells (MSCs) are used extensively for multiple indications in regenerative medicine. They are highly heterogeneous in terms of their biological characteristics and their mechanisms of action, making standardization a challenging undertaking. Furthermore, the use of MSCs in therapy appears to attract diverse views, ranging from concern and caution to enthusiastic positivity. We conducted semi-structured interviews with 20 expert stakeholders from academia, industry, regulatory agencies, non-governmental organizations and clinicians to explore their views, experiences, recommendations, and concerns regarding standardization of MSCs. Qualitative thematic analysis of transcribed records led to development of a consensus framework, which identified 5 key themes to facilitate exploration of the interviews’ content.

On the basis of our findings, we conclude that (1) there is undoubtedly an appetite for standardization, particularly in development of assays that enable comparison or benchmarking across manufacturers, processes, and cell sources; (2) stakeholder groups are not homogeneous in their concerns and attitudes; (3) careful consideration must be given to the points along the development timeline at which different standardization approaches could be beneficial; and (4) the roles of standards could be promoted further for specific aspects of advanced therapy medicinal product (ATMP) development and regulation such as qualification of decentralized manufacturing sites. A unified cross-stakeholder approach will help to advance MSC therapeutics and other cell therapy medicines.

## Graphical Abstract

**Graphical Abstract:**
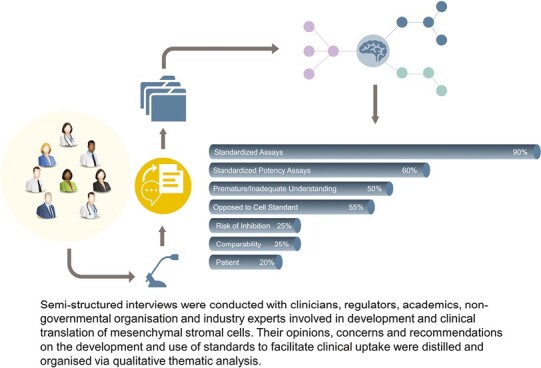

**Significance Statement**

This study represents a unique approach to assessing the issues around standardization of mesenchymal stromal cells (MSCs). It explores the views of a range of stakeholders involved in clinical translation of MSCs and analyses their concerns and recommendations to clarify opportunities and uncertainties associated with standardization. The study also identifies several recommendations that should be considered by standards and regulatory bodies to maximize the benefits of standardization, and specific areas in which standards could be better promoted to facilitate translation of MSCs into routine clinical use.

## Introduction

Mesenchymal stromal cells (MSCs) have been explored in numerous clinical indications based on immunomodulation via live^1^ and apoptotic cells,^2^ trophic repair effects^3,4^ and novel mechanisms such as mitochondrial transfer^5^; direct differentiation into de novo tissue^6^ has largely been discounted.^7,8^ The biology of MSCs is complex and dynamic; their characteristics are impacted by differences in tissue source, isolation, and culture conditions.^9-11^ Heterogeneity is widely recognized^12^ even within clonal populations^13-15^ and is often overlooked where the label “stem” is applied, leading to unrealistic expectations of therapeutic benefit.^16,17^ Heterogeneity presents particular problems in the context of regenerative medicine: comparability and consistency are extraordinary challenges to the approvability of MSC-based therapies.

Advanced therapy medicinal product (ATMP) developers identify a lack of standards as a significant barrier to progress.^18^ They are essential to lower research and development costs^19^ and can impact the entire value chain.^20^ Cell therapy product standards are seen as critical to patient safety as well as development of the field^21^ and are the subject of considerable effort within the International Standards Organization (ISO).^22^ The International Society for Cell and Gene Therapy (ISCT) position paper^23^ is frequently referenced as a characterization benchmark.^24,25^

Although many publications have called for standardization activities around cell therapy translation,^21,26,27^ they tend to be individual perspectives from single authors or teams. The authors highlight the need to develop standard assay methods and treatment protocols, production processes, and even standardized cell specifications. There is recognition that the field needs a range of tools to address the complexities inherent in the translation of such a heterogeneous cell type and that developing individual solutions in isolation will not facilitate overall progress toward realizing the clinical potential of MSCs. This study analyses a range of opinions from across the cell therapy field and brings together multiple viewpoints and perspectives. It was intended to identify specific areas in which standardization could be most beneficial to different groups and aspects that may present particular difficulties in terms of content, adoption, and utility. Against this background of ongoing interest in development of standards for MSCs, we conducted semi-structured interviews with 20 stakeholders from academia, industry, regulatory agencies, non-governmental organizations (NGOs) and clinicians to explore their views, recommendations, and concerns. Our research identified clear support for the development of standardized assays, raised specific concerns regarding standardization of MSCs themselves which should be addressed in future standards development, and also highlighted heterogeneity of opinion within stakeholder groups.

## Methods

### Ethical Approval

Ethical approval including approval of study documentation and informed consent was obtained under the University of York’s research ethics framework.

### Participants

A purposive sampling approach^28^ was taken given the specific expertise needed for the subject matter. The researchers’ own experience in the field was used to identify potential respondents from clinicians, academia, industry, regulatory agencies, and non-governmental institutions.

### Interviews

A workflow was developed to ensure consistency of approach and guide the practical aspects of the interview process (Supplementary Fig. S1). Interviews were conducted and recorded via video-conferencing platforms, each taking between 30 and 45 min. Transcripts were reviewed against audio files and edited to create “corrected transcripts” by identification of speaker (respondent or interviewer), removal of repetition, and correction of mistranscribed technical language.

### Analysis

#### Sentiment Analysis

Sentiment analysis seeks to identify emotional content in written text, using natural language processing to identify and score words and sentences indicative of positive and negative feelings.^29^ This approach was chosen to explore whether respondents’ language suggested very strong or outlier opinions and was assessed in 2 ways. First, using the Bing lexicon,^30^ which classifies individual words as positive or negative. Second, sentence sentiment was scored using the *sentimentr* package^31^ with the Jockers-Rinker lexicon^32^ which modifies sentiment according to context, using proximate words that convey negation (*not, can’t*) and intensity (*absolutely*, *certainly*, *almost*, *barely*) to adjust the sentiment score for that word. Text processing and sentiment analysis were undertaken in R^33^ with the *tidytext* package.^34^

#### Qualitative Thematic Analysis (Nvivo)

The main focus of this research is exploration of opinions and ideas around standardization using qualitative thematic analysis.^35^ This allows identification of themes or concepts in content, and organization to facilitate interpretation and analysis rather than simply summarizing data.^36^ Our approach was based on Burnard,^37^ with the analysis of corrected transcripts and organization of resultant themes undertaken using Nvivo Release 1.6.1 (QSR International), a package designed for qualitative or mixed-methods research involving unstructured text and other non-numerical source material. Data were categorized by combining concept-driven development of “codes” (relevant keywords or phrases) and data-driven iterative organization of codes, as described by Kuckartz.^35^

#### Development of Coding Structure

**Figure 1.**
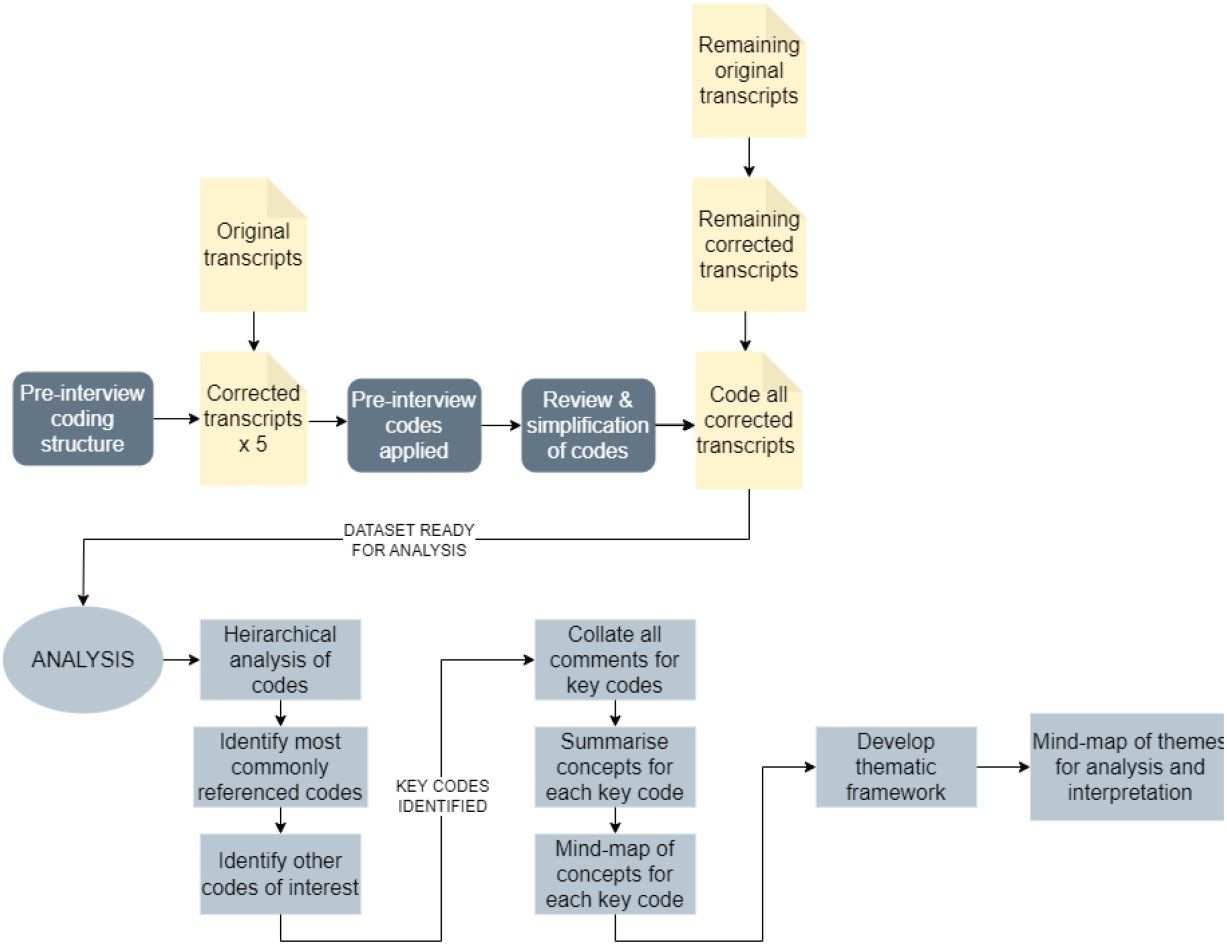
Workflow for the processing of interview transcripts and development of the thematic framework for analysis of the data. Prior to analyzing the interview transcripts, a series of “codes” (key words or phrases relevant to the subject), was prepared. An initial group of 5 corrected transcripts was “coded” in Nvivo by labeling (highlighting) each reference by a respondent to a specific code. These 5 initial coded transcripts were reviewed to assess the suitability of the initial list of codes, allowing elimination of duplicate, or closely overlapping codes. All transcripts, including the 5 initially used to review the code list, were then coded against the final set of codes. Hierarchical analysis identified the most frequently mentioned codes; these were then examined using mind-mapping to develop the overall thematic analysis.

A prospectively defined set of codes reflecting likely interview content was used to code 5 corrected transcripts. This involves tagging (highlighting) each mention of a code in the corrected transcript, allowing Nvivo to identify and organize interview content by code. These 5 transcripts were then reviewed to assess the suitability of the initial codes, allowing the elimination of unused or closely overlapping codes. All transcripts, including the first 5, were then coded against the final set of codes (Fig. 1).

#### Development of Thematic Framework

The most frequently referenced codes were analyzed to identify recurring themes and concepts common to all or most respondents using Nvivo’s code mapping functions. All references in the dataset to each of these “key codes” were then tabulated manually and one or more short themes or concepts were annotated against each reference. These short themes were grouped and “mind-maps” were prepared to allow visualization of the overall output for that code (Fig. 1). An overall thematic framework was prepared to facilitate exploration of the comments, concerns, and opinions arising from the interviews.

## Results

### Responses to Interview Request

Fifty-one potential respondents were contacted: 17 (UK), 14 (US), 4 (Canada), 4 (Ireland) 2 (Spain), and one each from 10 other countries. Respondents were identified by their primary area of interest; for example, research doctors actively involved in patient treatment/clinical trials were recorded as “clinician” rather than “academic”; academics working in a commercial capacity were assigned to the “industry” group.

**Figure 2.**
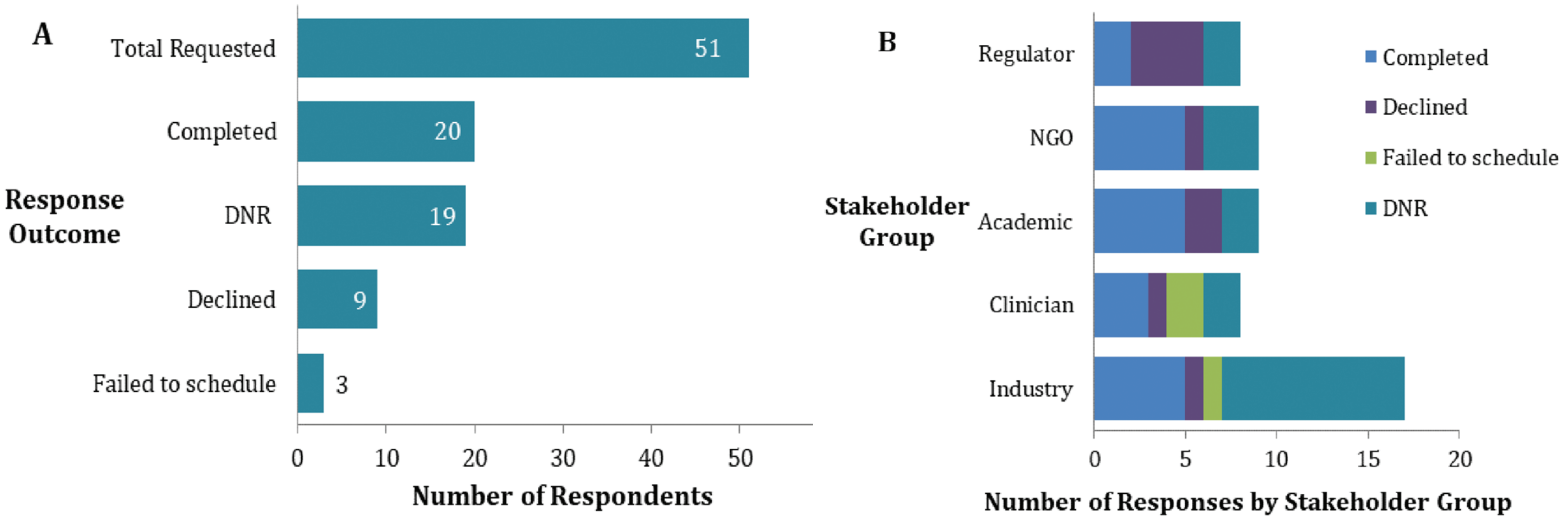
Disposition of respondents. (**A**) The numbers of potential interviewees who agreed and were interviewed (“Complete”) and who declined (“Declined”) or did not respond to the invitation (“DNR”). Where a respondent initially agreed to take part but did not schedule/attend the interview this was recorded as “Failed.” (**B**) The number of responses broken down by stakeholder group: academic, industry, regulatory agency, clinician, or NGO.

Selection of potential respondents was initially based on the researchers’ knowledge of the field. A second group was identified based on published activity in the MSC/standardization/regenerative medicine areas. Of these 28 “cold call” invitations 18 did not respond to our request. Of the 10 who did, 4 agreed and were interviewed. Once the target of 20 interviews had been achieved no further invitations were made. Responses and stakeholder field are summarized in Fig. 2.

### Sentiment Analysis

**Figure 3.**
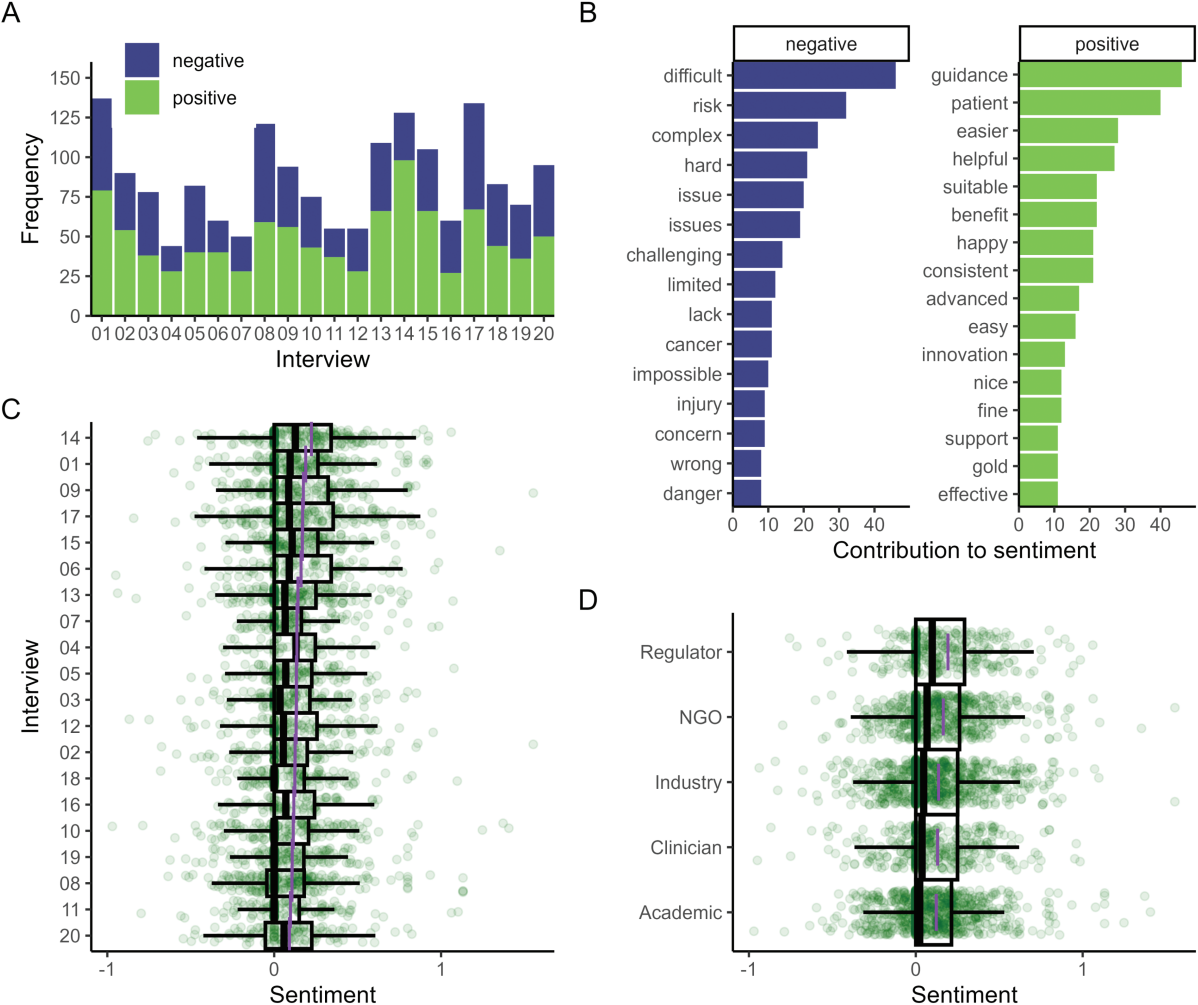
(**A**) Frequency of words spoken by each respondent that are classified as positive or negative in the Bing lexicon. (**B**) Contribution made by different words to the overall positive/negative sentiment across the entire corpus. The words “critical” and “isolate” were removed from the list of negative words. (**C**) Average sentiment of words for each respondent; the score for each word is modified by its proximity to words that convey negation (not, can’t) and intensity (absolutely, certainly, almost, barely). (**D**) Average sentiment of words for each category of respondent, modified as in (C). In C & D each green dot represents the sentiment-adjusted score for an individual word. The purple lines represent the mean word score for all words used by that respondent/respondent group. The box-and-whisker plot overlay indicates the median word score and the inter-quartile range (IQR) and extends to ± 1.5IQR. The apparent thick green vertical line at 0 in each sentiment score (Fig. 3C,3D) is an artifact reflecting overlapping scores of a large number of words all having a score of 0. The small range of the x-axis reflects the limited strength of sentiment–few words exceeded an overall score of either −1 or +1.

Respondents’ use of words associated with positive or negative emotions (Fig. 3A) indicates that in general, slightly more words with positive connotations than negative words were spoken by each respondent. The most frequent words used which contributed to the overall positive/negative sentiment (Fig. 3B) are shown, with concepts around difficulty, risk and complexity contributing most to the negative sentiments. Positive sentiments included guidance, ease and help.

Overall sentence sentiment is shown for each respondent (Fig. 3C) and by stakeholder group (Fig. 3D).

A text mining approach^38^ was used to explore the frequency of word stems (unigrams), pairs of words (bigrams), and triplets (trigrams) used across all respondents and by stakeholder group. Frequency charts were generated using R (Supplementary Figs. S4–S6) and by the respondent group (Supplementary Figs. S7–S9) to visualize the language used by the interviewees.

### Qualitative Thematic Analysis

#### Development of Coding Structure

Initially, 60 codes (items discussed by respondents) were prepared prior to interviewing. Five corrected transcripts were coded to assess the relevance and completeness of these initial codes. Nvivo code frequency analysis highlighted unused codes and manual review identified those that effectively duplicated another code. Thirteen were deleted leaving 47 codes.

#### Thematic Analysis Structure

**Figure 4.**
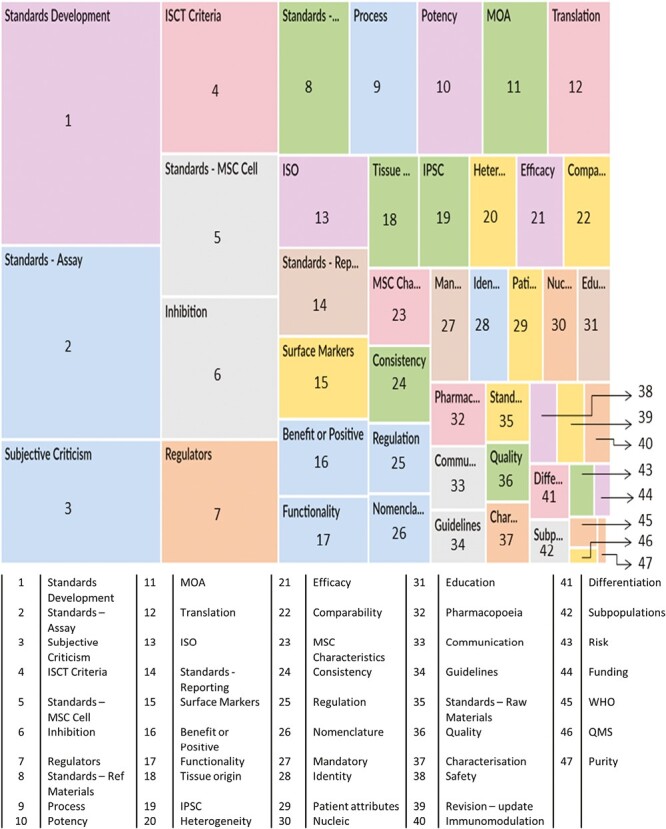
Hierarchy chart—most frequent items discussed by respondents by a number of coding references. The chart is generated by Nvivo from the total coding for all 20 interview transcripts, based on the numerical frequency with which each subject area was discussed by the respondents overall.

The most common codes are represented as a hierarchy chart (Fig. 4). “Standards development” was the most widely discussed element. This code included aspects such as the process of development, timescales for production, and the involvement of different stakeholders in the process of generating and promoting standards. Standardized assays were also discussed extensively and were widely favored (see also Fig. 6).

Most respondents discussed the ISCT criteria, either specifically using this term or by inference (eg “we use the standard marker panel”) which the researcher then explored to confirm that they did mean the ISCT panel. The concept of a standard set of requirements for MSCs (a cell specification) was frequently mentioned, as were concerns that standards could inhibit or adversely impact development or translational activities. Different types of standards arose frequently, with all but one (specific standards for raw materials) appearing in the top 20 categories. Note that this figure highlights the extent to which different aspects were discussed but does not indicate whether respondent views were positive or negative.

**Figure 5.**
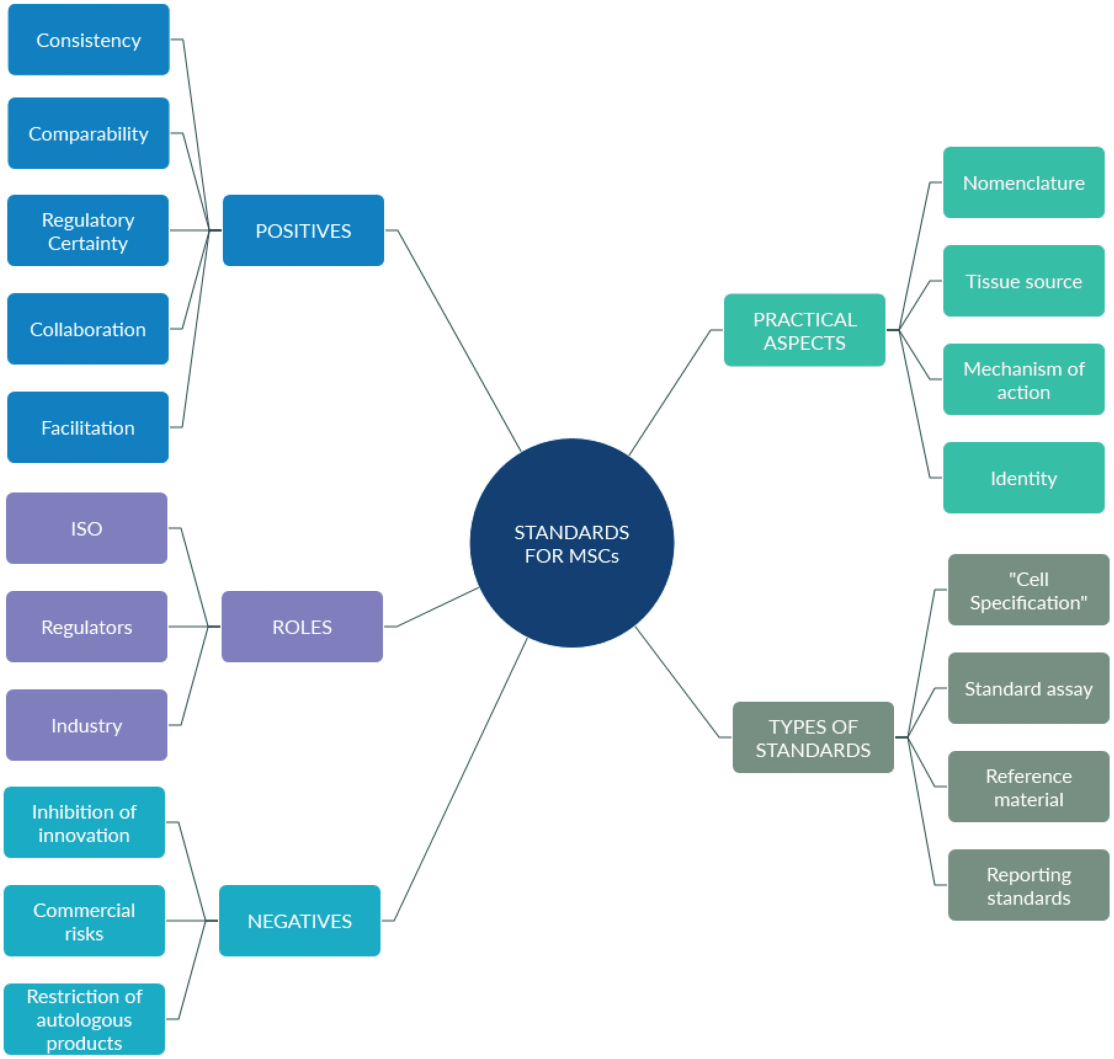
Overall thematic framework. The project distilled the themes around standardization of MSCs into 5 areas: potential benefits of standardization, potential concerns, and disadvantages, the types of standards that could be developed, the roles and involvement of various stakeholders, and practical issues to be considered.

The content for each code was collated manually by tabulating each comment, summarizing it into 1 or 2 themes, for example, “research culture,” “stakeholder involvement,” and these themes were then mind-mapped to produce a visualization of the content around each code. The interview content is condensed into 5 main themes: benefits of standardization, concerns or negatives, types of standards that could be beneficial, roles of stakeholder groups in the development and adoption of standards, and practical aspects relating to the complexity of MSCs. An overall thematic framework was prepared to capture the outcomes of the study (Fig. 5).

Given that this study is qualitative and focuses on respondent opinions, the results include individual quotes chosen to highlight specific points. Consistency and comparability were commonly highlighted as potential benefits of standardization, both from manufacturing and clinical/patient perspectives.

*Clinician 2: “Whenever I’m treating patients, making sure that, you know, each patient is getting the same therapy, and the confidence that if I do a trial, and show cell X works. And if I’m giving cell X, in the future, I want to make sure that batch is equally effective.”*

The importance of comparing results across studies was mentioned by all groups, either directly or in noting that absence of standards made such benchmarking extremely difficult, and this comparison is exacerbated by the recognized heterogeneity of MSCs.

*Industry 1: “At the moment there’s absolutely no way to benchmark against other studies, because you literally don’t know what the cells are, and what we know is that the origin makes an enormous difference so obviously a bone marrow mesenchymal cell is not the same as adipose mesenchymal cell is not the same as one from umbilical cord.”*

Interviewees with a more sophisticated regulatory perspective also mentioned the importance of comparability in facilitating use of newer licensing concepts such as decentralized manufacture:

*Industry 5: “If they would accept it [decentralized manufacture] based upon standardization, it would make things a lot easier, and I know a lot of companies would be very interested in that kind of model of decentralized manufacturing, because it makes the supply chain, the logistics chain of the process of manufacturing so much easier. So, if you could introduce a set of standards that will allow the acceptance of that decentralized manufacturing to become easier and smoother, it will definitely be attractive to industry.”*

It was suggested by NGOs involved in facilitating collaborations at the interface between academia and industry that non-mandatory standardization could benefit aspects of early academic work, particularly reproducibility and record-keeping.

*NGO 1: “The advantage for a research group in adopting work practices which are industry compliant at the late stage of their research is that, in theory anyway they should be able to cut out most of the development steps if they hand off as part of an exit strategy for the technology. Because all that needs to be done … is the thing needs to be replicated batch on batch in large numbers. So, that means (a) you access market quicker and maximize your patent lifetime usage and (b), it means that you’re more likely to be adopted, if you want to sell to big pharma or somebody else, because it’s all ready to go, and therefore you have credibility with people who are coming in with that mindset.”*

The imposition of formal standards for MSCs could be inhibitory to innovation and development of ATMPs tailored for specific indications. Academic respondents in particular expressed reservations and emphasized the need for flexibility to avoid negative impacts on research culture: researchers could resent or reject what might be perceived as unnecessary restrictions on their activities.

Several respondents raised a concern that MSC product standards could result in products that were simply compliant rather than being optimized for specific indications and stressed the importance of avoiding assumptions around what might constitute the “best” MSC. This idea was related to a significant concern regarding the extent of understanding of MSC biology, and that standardization of MSC products is premature given, in particular, the ongoing difficulties in even defining an MSC. One regulator drew a parallel with development of mobile phone technology:

*Regulator 2: “So to be almost the equivalent of nailing your colors to the mast for the mobile phone that’s at 1G or 2G or something like that, and then that would actually become counterproductive and prevent future development.”*

The existence of a cell standard may inadvertently create the impression that we know more than we do, thereby indirectly posing a risk to innovation:

*Academic 2: “I see the risk that people would imagine that if there is a proposed standard then everything is basically understood, we just need to comply with a standard and it will work. And it’s not like that we know, and even if there will be a proposed standard at a certain point, it will ­continuously have to be further developed, refined, confirmed, adapted maybe to a specific category of patients that require a different particular delivered signal by MSCs than another category of patients, even within the same indication. So, the risk of the standardization is to generate closed views, dogma-like conceptions, and that is a risk for the field.”*

At least one stakeholder from each group clearly opined that our understanding of MSC biology is immature, in particular regarding mechanisms of action driving expected therapeutic benefits.

#### Roles and Involvement of Stakeholders

There was a strong sense that no particular stakeholder group holds the key to successful standardization or indeed successful translation of ATMPs. Standardization could be a double-edged sword: are we giving our hard-won knowledge away for the benefit of others? Or conversely can we set the bar high enough to discourage competition? Impeding competition may be a benefit to some but surely would be a negative for the ultimate beneficiary, the patient.

Involvement in standardization activities as a means of influencing the development of the field, or to avoid being blindsided by new and unexpected requirements came over as a clear positive from both NGOs and regulators. This is unsurprising given that these stakeholders are most likely to have an appreciation of the purpose of standardization, and also to have practical experience of standards generation.

Regulator 1: *“And I think that we need to push for, you know, this education of people that actually, they could be shaping up the future with the knowledge that they’re generating and by participating in these standardization work streams”*

Industry and academic respondents favored engagement in standards development, largely rejecting the suggestion that this might entail handing over proprietary knowledge “for free.” The idea of cross-stakeholder standardization was supported, tying into the idea that any positives would benefit the whole field. While larger companies were considered suitable to lead standards development it was noted that they may perhaps reap proportionately fewer advantages because of their familiarity with regulatory requirements:

*NGO 2: “You know the big companies have the benefit of the subject matter expertise, the knowledge, the critical mass. What’s interesting is most companies, most big companies want to know how standards fit their processes as opposed to the other way around, small companies who don’t have either the critical mass experience or expertise are looking for guidance.”*

Conversely, standardization of processes, equipment, materials, and assays was mentioned as a benefit for larger companies that could leverage economies of scale when developing more than one product.

The importance of regulators’ engagement was frequently mentioned, although there was recognition that standards would be secondary to extant regulation rather than an alternative approach.

*Industry 1: So, if we can find a set of standards that are internationally acceptable that don’t interfere with the local regulatory requirements and don’t supersede or undercut those. That would be phenomenally useful.”*

*Industry 2: “Ultimately, it’s the interaction with the regulators that trumps everything.”*

There are real concerns about the length of time to prepare a standard followed by adoption and uptake by target audiences, which could create a state of perpetual obsolescence. One academic was concerned that attempting to gain consensus quickly might lead to a “lowest common denominator” standard:

*Academic 6: “The other side is that if the bar is too low, which is something that I’m very worried about, then you get all of these suspect clinics laying claim to legitimacy, based on adherence to extremely low bar standards that are really not standards. And that legitimizes their work and their research, and I think, for the most part, patients especially are not able to decipher that and if something looks like it’s an ISO standard or has that kind of stamp of approval, I think there’s a great danger that you’re promoting and allowing bad actors into this.”*

**Figure 6.**
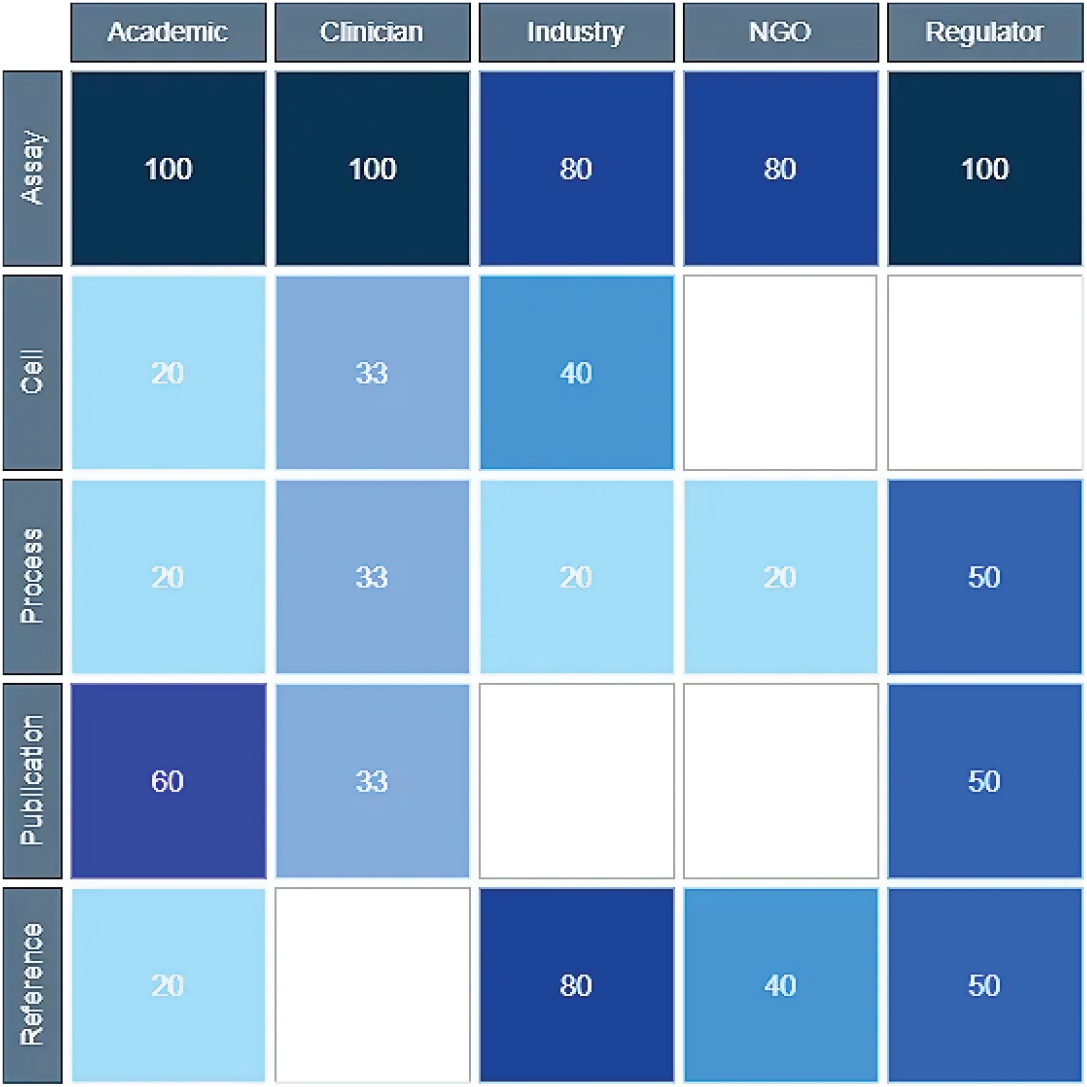
Respondents expressing a positive view of different types of standards that could be beneficial for MSCs. For each standard type, the number of respondents making positive comments was collated, and then grouped by stakeholder group. The proportion of positive comments is expressed as a percentage of the total respondents within each stakeholder group.

The interview guide included questions on what types of standards could be beneficial. Standardized assays were widely viewed as comparatively low-hanging fruit (Fig. 6).

Potency assays represented very important benefits: inter-batch consistency, comparability between clinical trials and/or manufacturers, benchmarking in relation to clinical outcomes, and transparency of published literature. The enthusiasm for standard potency assays was tempered with caution regarding insufficient understanding of biology and therapeutic activity; most respondents saw the development of potency assays as at once extremely challenging and vital to the progression of the field.

*Regulator 1: “I think the biggest challenge that the cell therapy community faces, is the lack of potency assays or the lack of specific assays that can let us know how potent a cell-based product will be, and that emerges because we don’t know enough about the biology of the processes but it is all linked. So, in a way, we need to start with the basics, we need to establish these very simple standards that can help people just with the initial standardization. And the ISCT paper I think it has been critical or instrumental in, at least, making people test for the same thing.”*

Academic responders expressed strong support for minimal standards for reporting clinical trials. These are world-leading researchers who frequently undertake peer reviews for high-impact clinical and cell biology journals, and they expressed considerable frustration that articles are published without even minimal data on cell identity and characterization in clinical trials.

*Academic 1: “And I think a description of how you derived your cells, how you’ve characterized them and how they compare to other cells, short but critical, should be an absolute requirement, certainly for any clinical study. We were talking about biological studies, also for in vitro studies, in other words, not saying you must do it like this, but rather saying, show us that you thought about it and show us why you’ve done it the way you’ve done it and made the case. And if that became a standard, I think that would be transformative…”.*

All bar one academic respondent was strongly opposed to the notion of an “MSC specification” or standard for MSCs, again citing gaps in current knowledge as significant barriers to the production of such a standard.

*Academic 2: “So the concept of MSC standardization can be in my view rather misleading … So what I advocate and I think … is that the MSCs need to be characterized according to standardized assays… so it will be possible to compare whether preparation X for mode of action A is similar or not to preparation Y, with intended mode of action B. … And so in the end we will not have an MSC standard, we would have a gamut of different assays that will be introduced to characterize the MSCs and to define whether they can be released or not, for a very specific therapeutic goal.”*

## Discussion

This study was designed to explore concerns, recommendations, perceived benefits, and risks of standardization in regard to MSCs. Calls for standardization have arisen from multiple different researchers and groups: reference materials,^39^ identity,^16^ potency assays.^40^ The ISCT has made recommendations for identity, immunological characterization, immunomodulatory potency assays, and nomenclature for different tissue sources.^23,41–43^ As noted earlier,^22^ ISO has published several standards concerning biobanking and methods for MSC for research use. Despite the considerable volume of such publications, one of our most striking observations was that almost half of the respondents expressed concern that our understanding of MSC biology is insufficient to define cell standards. The ongoing discussions around nomenclature,^43^ difficulty in identifying criteria to distinguish MSCs from different tissues,^44,45^ and from other fibroblastic cells^46^ speaks to a wider uncertainty regarding mechanisms of action.^47–49^ These fundamental gaps in our understanding do represent a significant risk that premature standards or inappropriate scope may distort or inhibit the adoption of MSC-based therapies.

The quality of characterization data in MSC publications was emphasized: heterogeneity among MSC populations should necessitate detailed characterization and that journals could support the field by requiring minimal descriptive data to be included in manuscripts. This observation is consistent with our own research,^25^ in which we argue that introducing editorial standards for basic characterization could promote considerable improvements in understanding the true validity of MSC clinical studies.

Product standards could be especially problematic for autologous therapies given the inevitable variability in starting material. Challenges in setting release specifications could be amplified by imposition of external standards not based on the manufacturing capability for that specific product: one academic involved in the manufacture of autologous products emphasized that clinicians should be able to use out-of-specification product so long as it presents no harm to the patient. Conversely, another academic who has strong links to both clinical development and industry expressed the opposite view:

*Academic 1: “What matters is that those cells are not being implanted as a waste of time. You want to know that they have the capacity to do the job”*

Although superficially rather purist and unhelpful for the patient, this position recognizes that there are risks in the use of any ATMP, even autologous and that patients should only receive products having a reasonable expectation of efficacy. The balance between clinical judgment in an individual case versus the intention of regulatory and medical ethics frameworks (patients should receive safe *and effective* treatments) is a difficult one,^21^ but it highlights the importance of carefully evaluating the potential impacts of any standards as a mechanism for facilitating the development of cell therapies.

It is worth highlighting that the development of ATMPs as medicinal products is a special case in some regards. ATMPs are retained by academic groups and small spin-out companies to a much greater extent than more traditional products, which may be due in part to specificities in the regulation of these products in both the EU and the United States.^25^ This continuum of academic involvement in the development process results in a more heterogeneous audience for standardization. One respondent expressed considerable dissatisfaction when discussing the extent to which academia is involved:

*Academic 5: “I’m going to go out on a limb here now. And even though I am an academic myself, I feel that one of the reasons why this field is in the mess that it’s in is because it’s been in the hands of academics, and it should have been in the hands of industry experts who much better understand the idea of industrial standards, and the need for really carefully conducted specific tests so I think a lot of the waffle that we have in the field, wouldn’t be there if it had been driven by industry and you know I think it’s quite noteworthy that these committees that set these standards are all academics. So, if it were industry driven much more, I think we’d be better off. I’m sure that a lot of people who would be very annoyed to hear me say that but nonetheless that’s my opinion.”*

The idea that standards could inhibit innovative approaches and academic freedom was a strong theme. Clearly, researchers need freedom to follow lines of enquiry without being restricted by pre-defined requirements, although one respondent, an ex-academic with extensive industry experience, noted that mindset could be different in laboratories in which the goal is out-licensing a promising therapy rather than continual research. The balance between research freedoms and adoption of standardized aspects that facilitate reliable clinical outcomes is a difficult one requiring careful timing and will almost certainly be establishment-specific. However, an early appreciation within academia of the potential benefits of standardization should enable a timely progression to a more industry-ready development pathway.

Sentiment analysis indicated a slightly positive attitude to the discussion overall, although, perhaps inevitably given that respondents are professional scientists, the overall tenor of content was quite neutral. Sentiment analysis was explored as an additional dimension to the research, given that the small sample size makes between and within-group statistical comparisons impossible, and it offered some reassurance that there were no major outliers in the respondent pool in terms of attitudes.

The outcome of sentiment analyses can be influenced by choice of lexicon,^50^ and whilst several domain-specific lexicons have been published as data frames for R and other platforms^51^ none were found for scientific conversation. The lexicons used here scored some common scientific words as strongly negative: in particular “critical” is likely a signifier of importance, and “isolate” has no emotional weight whatsoever in the context of cell biology. We attempted to correct for this by manually removing the words “isolate” and “critical.”

Nvivo analysis is to an extent subjective. While it is very powerful at comparing code content and frequency, number of hits can be influenced by choice of what, and how much, text to include against a specific coding instance. So frequency is of limited value in determining popularity (importance) of content, and Nvivo was used as a starting point for organizing and developing themes within interviewees’ responses rather than analysis itself.

The study achieved 20 interviews. Sample size is a much-debated area that recognizes the information saturation point as a key criterion for study validity in qualitative research.^28^ The completion of 20 interviews compares favourably with some recommendations for sample size^52^ beyond which little new information is likely to be gained. The emphasis on an exploration of expert respondents’ concerns, opinions, and recommendations was mitigated against a simple questionnaire approach, which could have yielded more quantitative data but would not achieve the main aim of the work.

This study focused on MSCs because of their extensive clinical use, and because the extraordinary biological heterogeneity of MSCs presents particular challenges to standardization as a means of facilitating authorization and adoption into routine clinical practice. Our findings are also generalizable to the adjacent and expanding field of MSC-derived acellular therapies, which has now reached the clinical stage,^53,54^ and ATMPs more widely, particularly in the context of standardized assays and materials and in stimulating engagement of stakeholders both with the standards development process and with the adoption of standards in the development of their products.

## Concluding Thoughts

This research highlights not only differences in concerns and opinions between different stakeholders but also indicates heterogeneity of approach within groups. An innovator scientist with senior management responsibilities in industry viewed engagement with standards as something of a luxury and a potential distraction from the primary goal of product approval. Another industry respondent focused almost exclusively on the positives: simplifying operations and streamlining interactions with regulators. It may be that companies need to achieve a critical mass before they feel able to expend resources on standardization activities, and potentially these may be the ones who would benefit most from “off-the-shelf” guidance at an appropriate level such as standardized assays or materials.

It is important that we do not generate standards for standards’ sake, and those involved in drafting international standards might be encouraged to link standards development activities to specific opportunities such as decentralized manufacture or global licensing of allogeneic products manufactured in multiple regions. The relationship of standards to regulatory processes is not immediately apparent to many developers, especially academic spin-outs and small biotech companies. FDA has provided useful guidance on the acceptability of standards in applications to the Center for Biologics Evaluation and Research,^55^ which reviews applications for cell and gene therapy products. The ways in which standards can be leveraged in pursuit of a marketing authorization should be clarified by other regulators, particularly in the EU.

The interview process highlighted a lack of understanding of standards as an external benchmark in some respondents, who initially conflated standards with their own internal specifications or requirements. One important recommendation arising from this study is therefore that standards-generating organizations could consider how to promote the existence and the value of external standards to academic and small industry developers who do not typically engage with the standards development process and may not, therefore, be reaping the benefits of standardization.

On the basis of our findings (1) there is undoubtedly an appetite for standardization in specific areas, particularly the development of assays that can be used for comparison or benchmarking across manufacturers, processes, and cell sources, (2) stakeholder groups are not homogeneous in their concerns and attitudes, (3) careful consideration must be given to the points along the development timeline at which different standardization approaches could be beneficial, and (4) the roles of standards could be promoted further in regard to specific aspects of ATMP development and regulation such as qualification of decentralized manufacturing sites. Future development of this work could usefully explore the differences of opinion within stakeholder groups to inform development of more targeted methods of promotion of and engagement in standardization.

## Supplementary Material

szad056_suppl_Supplementary_Information_S1

szad056_suppl_Supplementary_Information_S2

## Data Availability

Data generated for this study consists of the unedited and corrected interview transcripts. Consent to be interviewed was given on condition of anonymity. Inevitably certain biographical information and references to current or prior positions are contained within the transcripts. Given the stakeholder context of the interviews, it is possible that some respondents’ identity could be inferred from the transcripts, therefore we do not intend to make the transcript content publicly available.

## References

[1] Miclau K , Hambright WS, Huard J, Stoddart MJ, Bahney CS. Cellular expansion of MSCs: shifting the regenerative potential. Aging Cell. 2023;22(1):e13759. 10.1111/acel.13759 36536521 PMC9835588

[2] Kholodenko IV , Kholodenko RV, Majouga AG, Yarygin KN. Apoptotic MSCs and MSC-derived apoptotic bodies as new therapeutic tools. Curr Issues Mol Biol. 2022;44(11):5153-5172. 10.3390/cimb44110351 36354663 PMC9688732

[3] Caplan AI , Dennis JE. Mesenchymal stem cells as trophic mediators. J Cell Biochem. 2006;98(5):1076-1084. 10.1002/jcb.20886 16619257

[4] Pabbruwe MB , Kafienah W, Tarlton JF, et al. Repair of meniscal cartilage white zone tears using a stem cell/collagen-scaffold implant. Biomaterials. 2010;31(9):2583-2591. 10.1016/j.biomaterials.2009.12.023 20053438

[5] Liu Z , Sun Y, Qi Z, Cao L, Ding S. Mitochondrial transfer/transplantation: an emerging therapeutic approach for multiple diseases. Cell Biosc. 2022;12(1):66. 10.1186/s13578-022-00805-7 PMC9121600 35590379

[6] Baba S , Yamada Y, Komuro A, et al. Phase I/II trial of autologous bone marrow stem cell transplantation with a ­three-dimensional woven-fabric scaffold for periodontitis. Stem Cells Int. 2016;2016:6205910. 10.1155/2016/6205910 27990164 PMC5136404

[7] Spees JL , Lee RH, Gregory CA. Mechanisms of mesenchymal stem/stromal cell function. Stem Cell Res Therapy. 2016;7(1):125. 10.1186/s13287-016-0363-7 PMC5007684 27581859

[8] Pittenger MF , Discher DE, Péault BM, et al. Mesenchymal stem cell perspective: cell biology to clinical progress. npj Regener Med. 2019;4(1):22. 10.1038/s41536-019-0083-6 PMC6889290 31815001

[9] Wilson A , Hodgson-Garms M, Frith JE, Genever P. Multiplicity of mesenchymal stromal cells: finding the right route to therapy. Front Immunol. 2019;10(1112):1-8. 31164890 10.3389/fimmu.2019.01112 PMC6535495

[10] Salerno A , Brady K, Rikkers M, et al. MMP13 and TIMP1 are functional markers for two different potential modes of action by mesenchymal stem/stromal cells when treating osteoarthritis. Stem Cells. 2020;38(11):1438-1453. 10.1002/stem.3255 32652878

[11] Marote A , Santos D, Mendes-Pinheiro B, et al. Cellular aging secretes: a comparison of bone-marrow-derived and induced mesenchymal stem cells and their secretome over long-term culture. Stem Cell Rev Rep. 2023;19(1):248-263. 10.1007/s12015-022-10453-6 36152233

[12] McLeod CM , Mauck RL. On the origin and impact of mesenchymal stem cell heterogeneity: new insights and emerging tools for single cell analysis. Eur Cell Mater. 2017;34:217-231. 10.22203/eCM.v034a14 29076514 PMC7735381

[13] Russell KC , Phinney DG, Lacey MR, et al. In vitro high-capacity assay to quantify the clonal heterogeneity in trilineage potential of mesenchymal stem cells reveals a complex hierarchy of lineage commitment. Stem Cells. 2010;28(4):788-798. 10.1002/stem.312 20127798

[14] James S , Fox J, Afsari F, et al. Multiparameter analysis of human bone marrow stromal cells identifies distinct immunomodulatory and differentiation-competent subtypes. Stem Cell Rep. 2015;4(6):1004-1015. 10.1016/j.stemcr.2015.05.005 PMC4471830 26070611

[15] Rennerfeldt DA , Van Vliet KJ. Concise review: when colonies are not clones: evidence and implications of intracolony heterogeneity in mesenchymal stem cells. Stem Cells. 2016;34(5):1135-1141. 10.1002/stem.2296 26840390

[16] Sipp D , Robey P, Turner L. Clear up this stem-cell mess. Nature. 2018;561:455-457. 30258150 10.1038/d41586-018-06756-9

[17] Wilson A , Webster A, Genever P. Nomenclature and heterogeneity: consequences for the use of mesenchymal stem cells in regenerative medicine. Regen Med. 2019;14(6):595-611. 10.2217/rme-2018-0145 31115266 PMC7132560

[18] ARM. Pharma and Biotech Survey. Washington DC, USA. 2014.

[19] Hunsberger J , Harrysson O, Shirwaiker R, et al. Manufacturing road map for tissue engineering and regenerative medicine technologies. Stem Cells Transl Med. 2015;4(2):130-135. 10.5966/sctm.2014-0254 25575525 PMC4303363

[20] McNiece IK , Wacker KK, Kurtzberg J, Warkentin PI. Standardization, workforce development and advocacy in cell and gene therapies: a summary of the 2020 Regenerative Medicine InterCHANGE. Cytotherapy. 2021;23(10):886-893. 10.1016/j.jcyt.2021.02.004 33775525

[21] Lomax GP , Torres A, Millan MT. Regulated, reliable, and reputable: protect patients with uniform standards for stem cell treatments. Stem Cells Transl Med. 2020;9(5):547-553. 10.1002/sctm.19-0377 32040254 PMC7180289

[22] Cao J , Hao J, Wang L, et al. Developing standards to support the clinical translation of stem cells. Stem Cells Transl Med. 2021;10(Suppl 2):S85-S95. 10.1002/sct3.13035 34724717 PMC8560191

[23] Dominici M , Le Blanc K, Mueller I, et al. Minimal criteria for defining multipotent mesenchymal stromal cells. The International Society for Cellular Therapy position statement. Cytotherapy. 2006;8(4):315-317. 10.1080/14653240600855905 16923606

[24] Mendicino M , Bailey AM, Wonnacott K, Puri RK, Bauer SR. MSC-based product characterization for clinical trials: an FDA perspective. Cell Stem Cell. 2014;14(2):141-145. 10.1016/j.stem.2014.01.013 24506881

[25] Wilson AJ , Rand E, Webster AJ, Genever PG. Characterisation of mesenchymal stromal cells in clinical trial reports: analysis of published descriptors. Stem Cell Res Ther. 2021;12(1):360. 10.1186/s13287-021-02435-1 34158116 PMC8220718

[26] Krueger TEG , Thorek DLJ, Denmeade SR, Isaacs JT, Brennen WN. Concise review: mesenchymal stem cell-based drug delivery: the good, the bad, the ugly, and the promise. Stem Cells Transl Med. 2018;7(9):651-663. 10.1002/sctm.18-0024 30070053 PMC6127224

[27] Shaw R. Illuminating the need for standards in regenerative medicine and advanced therapy. Cell Gene Therapy. 2021

[28] Vasileiou K , Barnett J, Thorpe S, Young T. Characterising and justifying sample size sufficiency in interview-based studies: systematic analysis of qualitative health research over a 15-year period. BMC Med Res Methodol. 2018;18(1):148. 10.1186/s12874-018-0594-7 30463515 PMC6249736

[29] Birjali M , Kasri M, Beni-Hssane A. A comprehensive survey on sentiment analysis: Approaches, challenges and trends. Knowledge-Based Sys. 2021;226:107134. 10.1016/j.knosys.2021.107134

[30] Hu M , Liu B, editors. Mining and Summarizing Customer Reviews. Tenth ACM SIGKDD International Conference on Knowledge Discovery and Data Mining; 2004 August 22-25, 2004; Seattle, US.

[31] Rinker T. Sentimentr: Calculate Text Polarity Sentiment Version 2.9.0. https://cran.r-project.org/web/packages/sentimentr/sentimentr.pdf.

[32] hash_Sentiment_Jockers_Rinker: Combined Jockers & Rinker Polarity Lookup Table. https://search.r-project.org/CRAN/refmans/lexicon/html/hash_sentiment_jockers_rinker.html.

[33] Team RC. R: A Language and Environment for Statistical Computing. Vienna, Austria: R Foundation for Statistical Computing; 2020. Available from: https://www.R-project.org.

[34] Silge J , Robinson D. tidytext: text mining and analysis using tidy data principles in R. J Open Source Softw. 2016;1(3):37. 10.21105/joss.00037

[35] Kuckartz U. Qualitative text analysis: a systematic approach. In: Kaiser G, Presmeg N, editors. Compendium for Early Career Researchers in Mathematics Education. Cham: Springer International Publishing; 2019. pp. 181-197.

[36] Maguire M , Delahunt B, eds. Doing a Thematic Analysis: A Practical, Step-By-Step Guide for Learning and Teaching Scholars. All-Ireland Society for Higher Education.2017.

[37] Burnard P. A method of analysing interview transcripts in qualitative research. Nurse Educ Today. 1991;11(6):461-466. 10.1016/0260-6917(91)90009-y 1775125

[38] Silge J , Robinson D. Text Mining With R 2022. Available from: https://www.tidytextmining.com/index.html.

[39] Viswanathan S , Keating A, Deans R, et al. Soliciting strategies for developing cell-based reference materials to advance mesenchymal stromal cell research and clinical translation. Stem Cells Dev. 2014;23(11):1157-1167. 10.1089/scd.2013.0591 24422625 PMC4027980

[40] Levy O , Kuai R, Siren EMJ, et al. Shattering barriers toward clinically meaningful MSC therapies. Sci Adv. 2020;6(30):eaba6884. 10.1126/sciadv.aba6884 32832666 PMC7439491

[41] Krampera M , Galipeau J, Shi Y, Tarte K, Sensebe L; MSC Committee of the International Society for Cellular Therapy (ISCT). Immunological characterization of multipotent mesenchymal stromal cells—The International Society for Cellular Therapy (ISCT) working proposal. Cytotherapy. 2013;15(9):1054-1061. 10.1016/j.jcyt.2013.02.010 23602578

[42] Galipeau J , Krampera M, Barrett J, et al. International Society for Cellular Therapy perspective on immune functional assays for mesenchymal stromal cells as potency release criterion for advanced phase clinical trials. Cytotherapy. 2016;18(2):151-159. 10.1016/j.jcyt.2015.11.008 26724220 PMC4745114

[43] Viswanathan S , Shi Y, Galipeau J, et al. Mesenchymal stem versus stromal cells: International Society for Cell & Gene Therapy (ISCT®) Mesenchymal Stromal Cell committee position statement on nomenclature. Cytotherapy. 2019;21(10):1019-1024. 10.1016/j.jcyt.2019.08.002 31526643

[44] Wegmeyer H , Bröske A-M, Leddin M, et al. Mesenchymal stromal cell characteristics vary depending on their origin. Stem Cells Dev. 2013;22(19):2606-2618. 10.1089/scd.2013.0016 23676112 PMC3780294

[45] Chen JY , Mou XZ, Du XC, Xiang C. Comparative analysis of biological characteristics of adult mesenchymal stem cells with different tissue origins. Asian Pac J Trop Med. 2015;8(9):739-746. 10.1016/j.apjtm.2015.07.022 26433660

[46] Robey P. “Mesenchymal stem cells”: fact or fiction, and implications in their therapeutic use. F1000Res. 2017;6:1-3. 10.12688/f1000research.10955.1 PMC5399967 28491279

[47] Bianco P , Cao X, Frenette PS, et al. The meaning, the sense and the significance: translating the science of mesenchymal stem cells into medicine. Nat Med. 2013;19(1):35-42. 10.1038/nm.3028 23296015 PMC3998103

[48] Martin I , Galipeau J, Kessler C, Le Blanc K, Dazzi F. Challenges for mesenchymal stromal cell therapies. Sci Transl Med. 2019;11(480):eaat2189. 10.1126/scitranslmed.aat2189 30787168

[49] Zhuang W-Z , Lin Y-H, Su L-J, et al. Mesenchymal stem/stromal cell-based therapy: mechanism, systemic safety and biodistribution for precision clinical applications. J Biomed Sci. 2021;28(1):28. 10.1186/s12929-021-00725-7 33849537 PMC8043779

[50] Hammer H , Yazidi A, Bai A, Engelstad P, eds. Building domain specific sentiment lexicons combining information from many sentiment lexicons and a domain specific corpus. In: 5th International Conference on Computer Science and Its Applications (CIIA); 2015; Saida, Algeria: Springer International Publishing;https://oda.oslomet.no/oda-xmlui/bitstream/handle/10642/3175/1249311nr2.pdf?sequence=1&isAllowed=y.

[51] Naldi M. A review of sentiment computation methods with R packages. *ArXiv*.2019;abs/1901.08319.

[52] Green J , Thorogood N. Qualitative methods for health research. In: Silverman D, ed. Sage; 2004.

[53] Warnecke A , Prenzler N, Harre J, et al. First-in-human intracochlear application of human stromal cell-derived extracellular vesicles. J Extracell Vesicles. 2021;10(8):e12094. 10.1002/jev2.12094 34136108 PMC8178433

[54] Sanz-Ros J , Mas-Bargues C, Romero-García N, et al. Extracellular vesicles as therapeutic resources in the clinical environment. Int J Mol Sci. 2023;24(8):7005. 10.3390/ijms24087005 36768664 PMC9917082

[55] FDA. Standards Development and the Use of Standards in Regulatory Submissions Reviewed in the Center for Biologics Evaluation and Research. 2019.

