# Supplementary material for: Attitudes Towards Standardization of Mesenchymal Stromal Cells—A Qualitative Exploration of Expert Views
Source: Stem Cells Transl Med. 2023 Sep 15;12(11):745–57. doi: 10.1093/stcltm/szad056 (PMC10630078; doi:10.1093/stcltm/szad056)
Supplement: szad056_suppl_Supplementary_Information_S1 [file szad056_suppl_supplementary_information_s1.docx]

**INTERVIEWS PAPER - SUPPLEMENTARY INFORMATION**

Contents

[1 Interviews Process Workflow 1](#_Toc137038747)

[2 Interview Guide 2](#_Toc137038748)

[3 Additional Analysis - Text Mining 3](#_Toc137038749)

# 1 Interviews Process Workflow


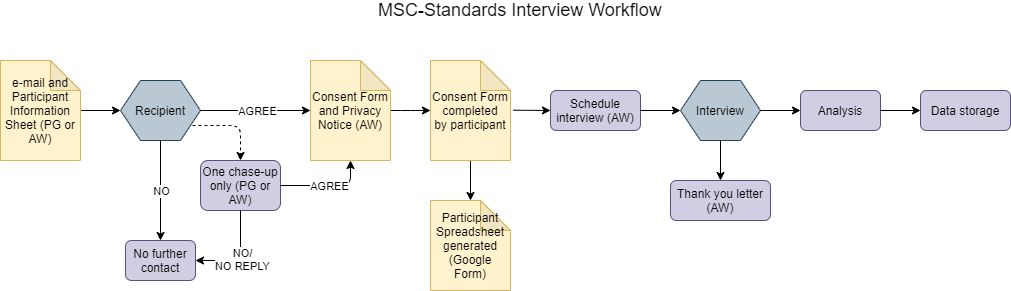


**Figure S1: Workflow for interviews process** highlighting the major activities and key documentation generated in conducting the research. Documentation generated and approved by the Ethics Committee within the Department of Biology included a Participant Information Sheet, provided to potential respondents prior to requesting consent; an informed consent form; a Privacy Notice setting out the respondents’ data protection rights under the UK General Data Protection Regulations 2018; and an interview guide (outline questions for the interviewer).

# 2 Interview Guide


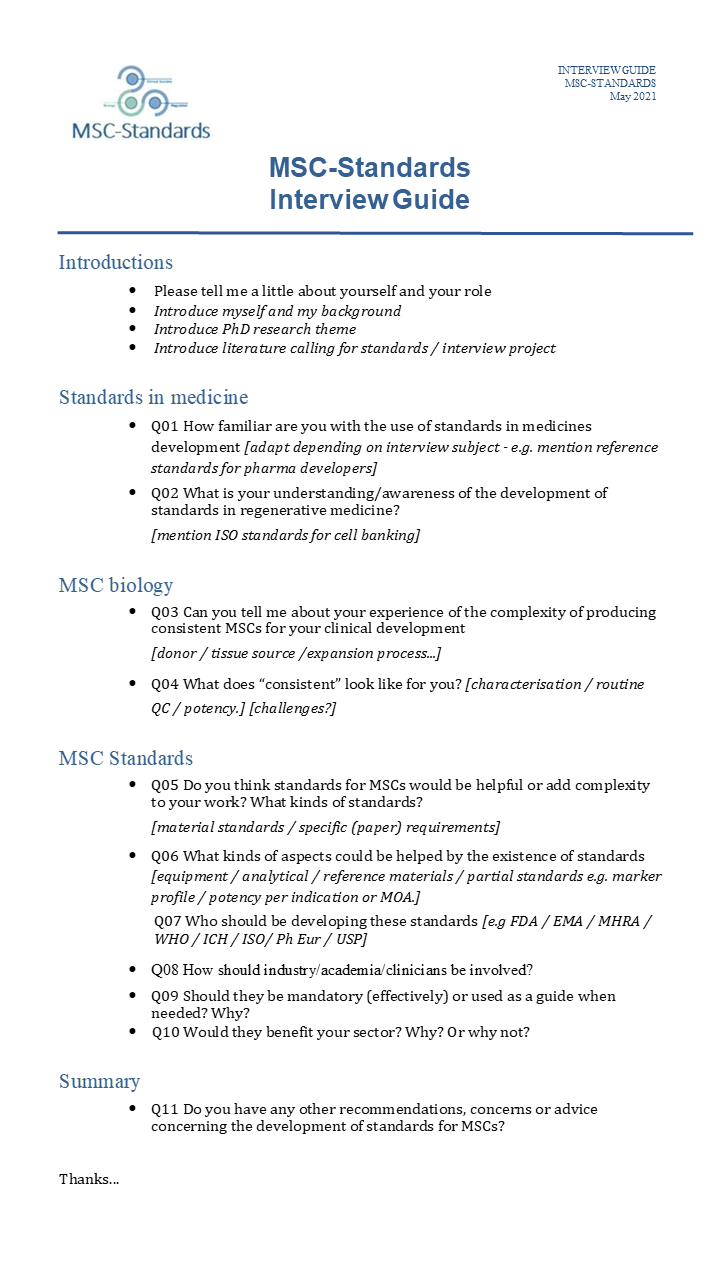


**Figure S2: Interview Guide.**

An interview guide was prepared and designed to provide some structure for the interview and as a prompt for the researcher, helping to keep interviews on track as the conversation proceeded. It was not intended to be used as a questionnaire and therefore not all questions were specifically addressed in all interviews. Some questions were not appropriate for particular interviewees, for example the questions on familiarity with standards were not directed to respondents from standards organisations.

# 3 Additional Analysis - Text Mining

A corpus containing all of the respondents’ words was prepared for this analysis. Text mining was undertaken in R. The processing of text though text mining in R beings with preparation of the dataset being analysed: the “corpus”. The corpus contains the content of the interview transcripts and can be interrogated at the single interview, the stakeholder group level or the all interviews level. The corpus is tokenized, a process which removes non-relevant elements of the corpus such as the white space between words, punctuation marks etc., and reduces the content to a set of word-level elements or “tokens” ready for conversion to “tidy” format (one token per row in the dataset) for further transformation and analysis in R. Tokenization by n-gram provides simple counts of single words stems (unigrams), bigrams (two words in conjunction e.g. “*surface marker*”, “*stem cell*”) and trigrams (three words in conjunction e.g. “*mesenchymal stem cell*”) [Silge & Robinson]. Stop words are frequently used words that add no useful information, such as pronouns, “the”, “and” etc. and are eliminated to avoid skewing of frequency analysis.

Stemming allows for conversion of tokens to stems such that frequency analysis will capture all token variants having the same stem within one count; for example, “*standard*” captures “*standard / standards / standardise / standardisation*”. Text mining counts the number of times a n–gram appears in a particular corpus. Results are displayed as a frequency chart with a pre-determined cut-off for lower limit of frequency of mentions across the corpus. N–grams are truncated by the stemming process.


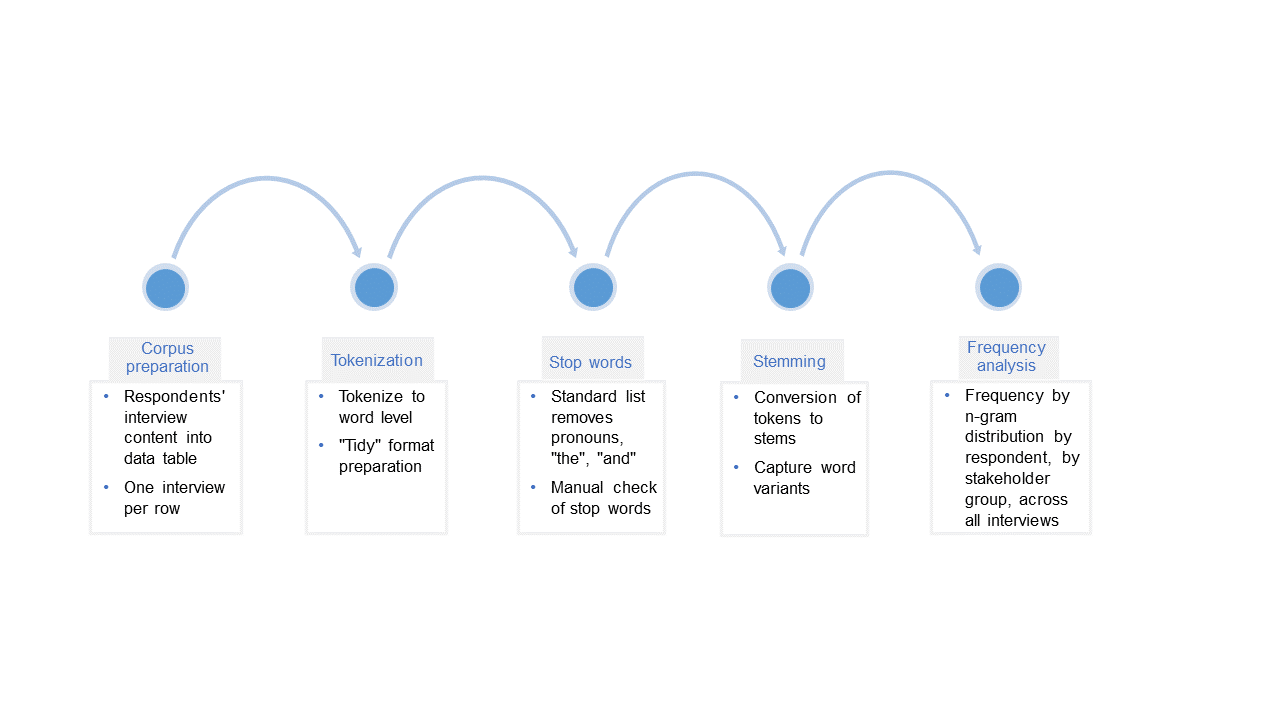


**Figure S3: Text Mining process steps, highlighting the process by which text mining is undertaken.**

Figures S4 – S9 illustrate distribution of word counts by uni-, bi- and tri-gram


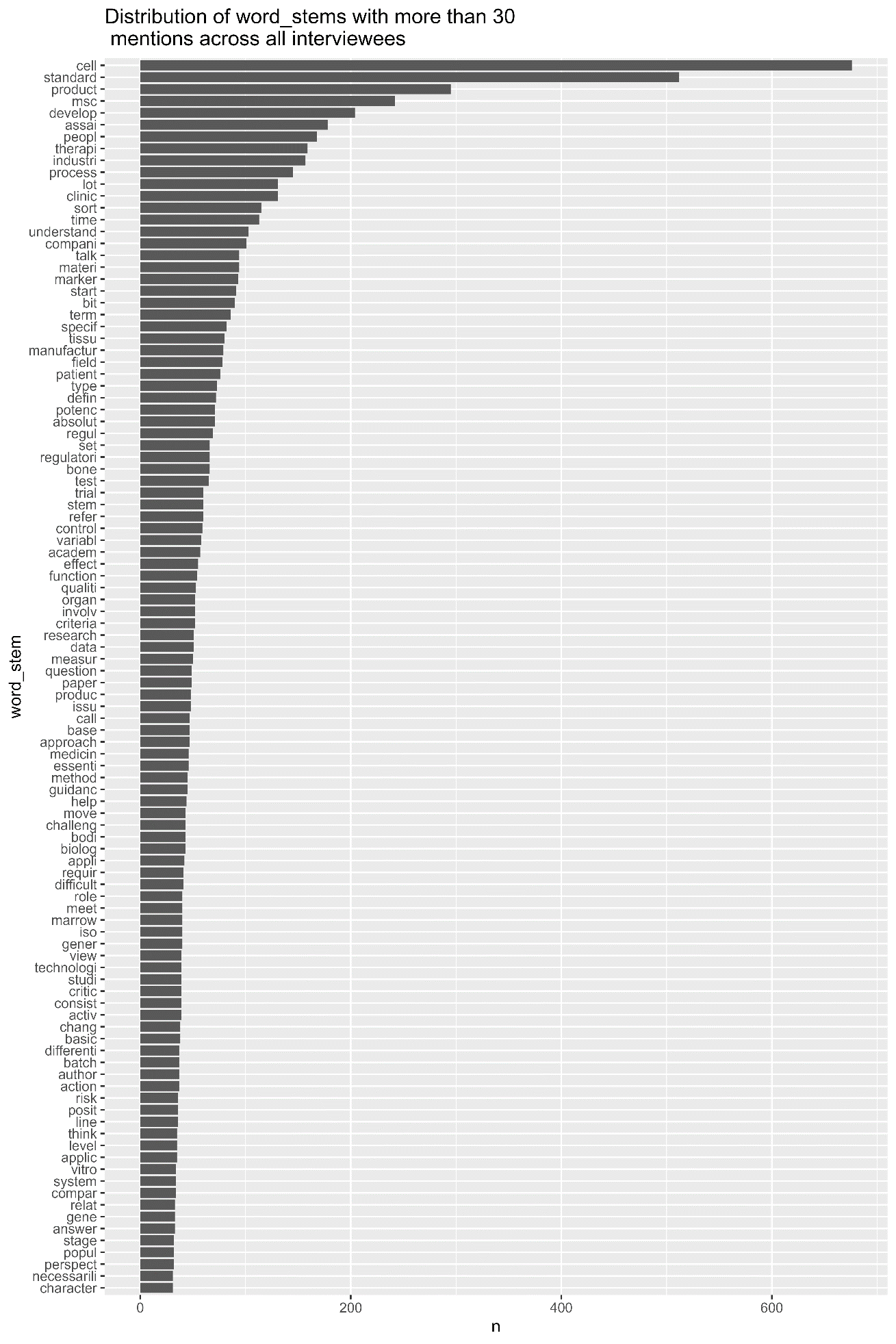


**Figure S4: Word count – stems with more than 30 mentions across all interviewees.**

A corpus containing all words used by each respondent in the interviews was prepared from the interview transcripts. Responses were tokenized into individual words (unigrams), and filtered to remove “stopwords” which are very common words not useful for analysis. Words were then “stemmed” to capture variants of single words. N–grams are truncated by the stemming process.


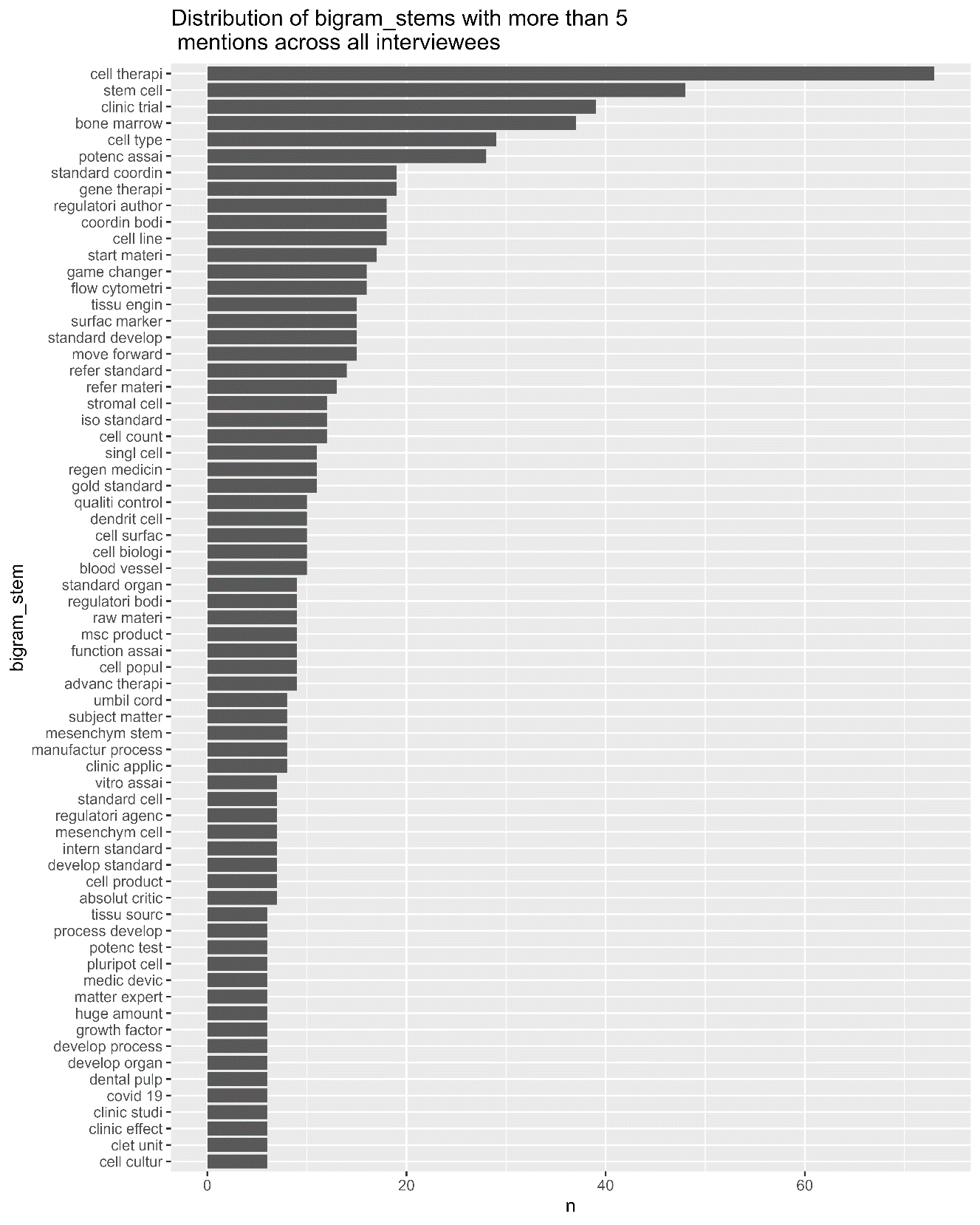


**Figure S5: Word count – bigram stems with more than 5 mentions across all interviewees.**

A corpus containing all words used by each respondent in the interviews was prepared from the interview transcripts. Responses were tokenized into adjacent pairs of words (bigrams), and filtered to remove “stopwords” which are very common words not useful for analysis. Words were then “stemmed” to capture variants of single words. N–grams are truncated by the stemming process.


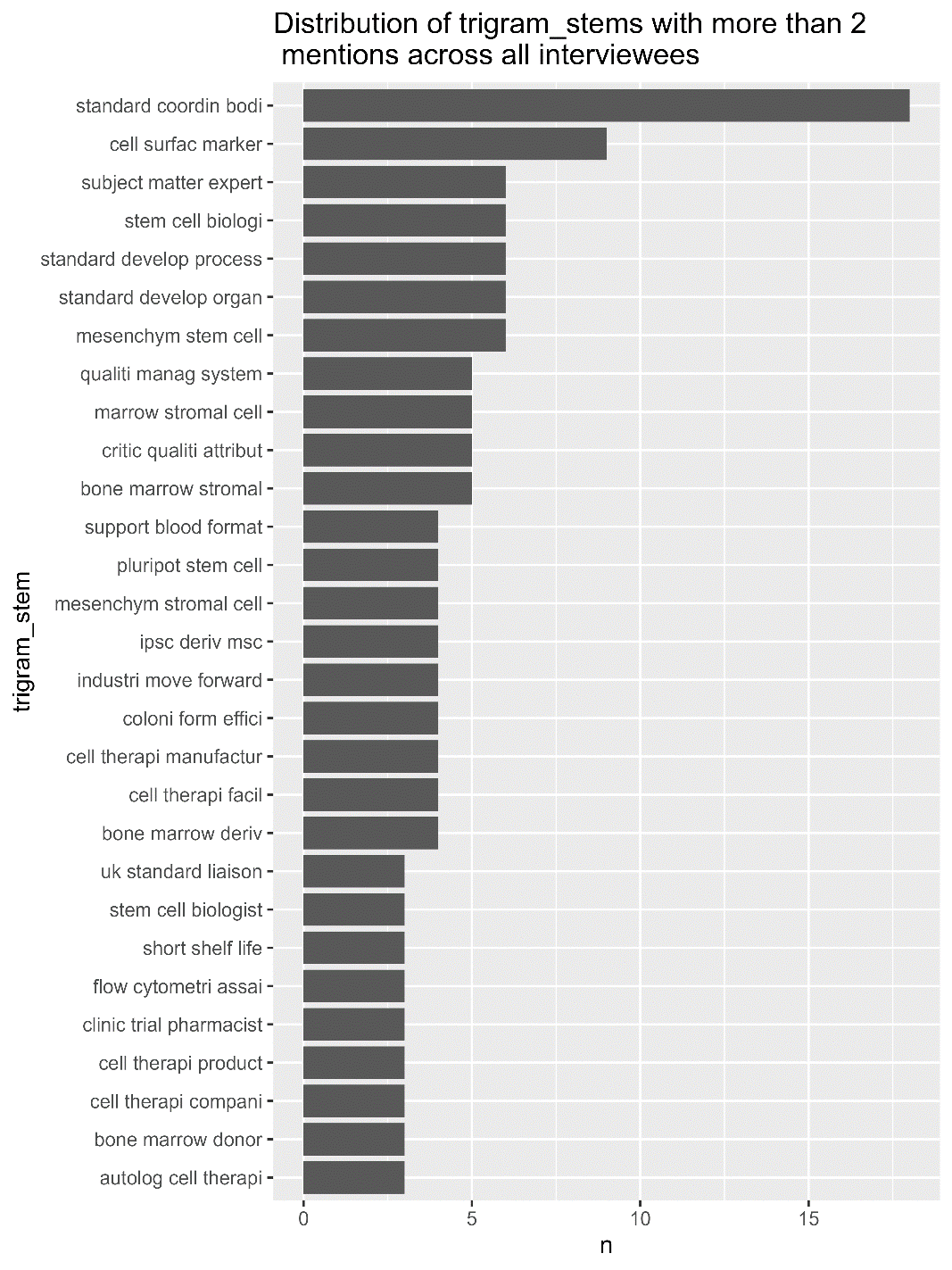


**Figure 6: Word count – trigram stems with more than 5 mentions across all interviewees.**

A corpus containing all words used by each respondent in the interviews was prepared from the interview transcripts. Responses were tokenized into adjacent sets of three words (trigrams), and filtered to remove “stopwords” which are very common words not useful for analysis. Words were then “stemmed” to capture variants of single words. N–grams are truncated by the stemming process.


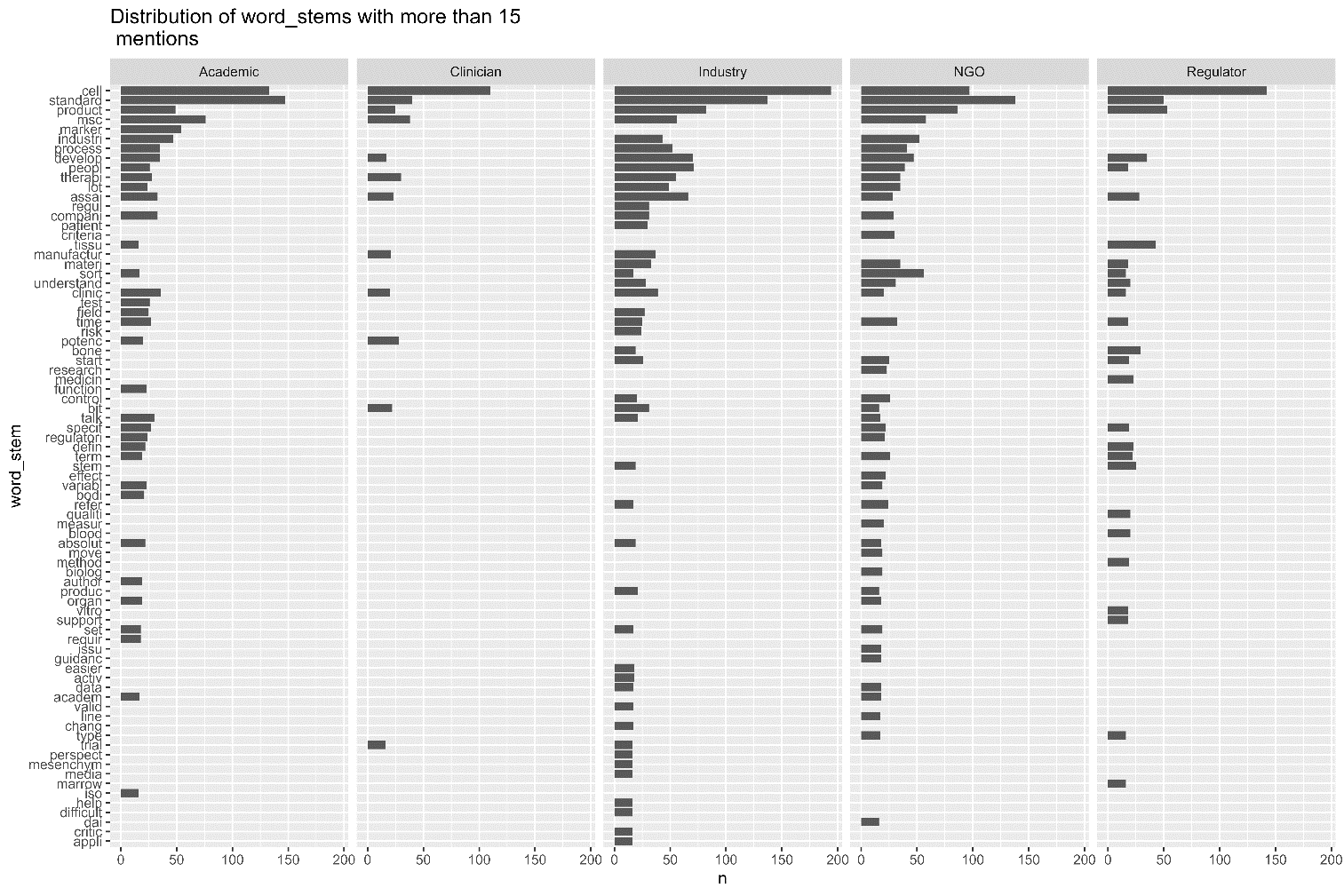


**Figure S7: Distribution of word stems with more than 15 mentions by respondent group**

The unigrams prepared for the previous counts are displayed by stakeholder group. It can be seen that the most common words occur with similar frequencies across the groups. Note that these are simple counts and are not normalised to the number of respondents in each group. Thus frequency counts are lowest in the clinician (n=3) and regulator (n=2) groups.


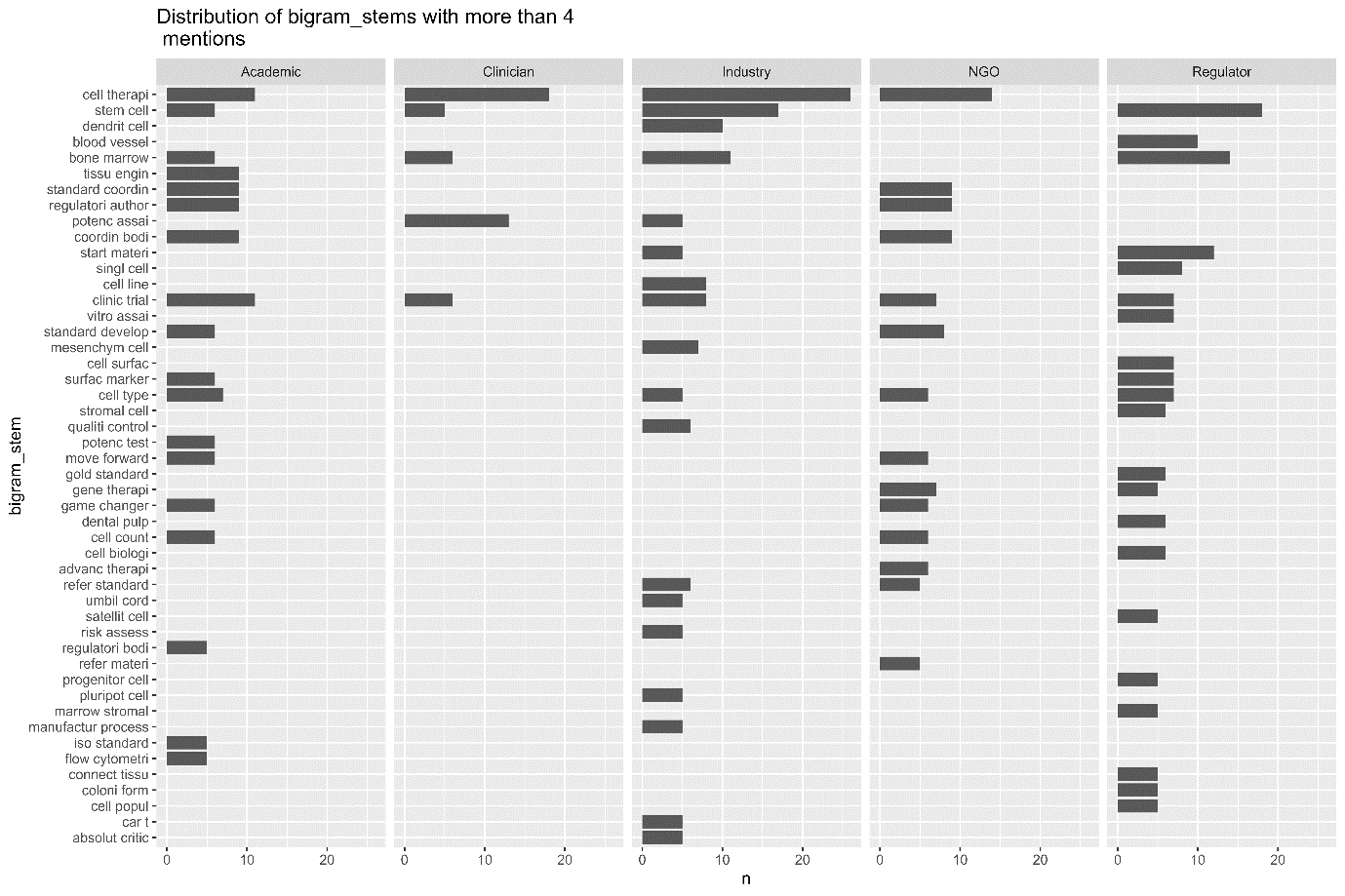


**Figure S8: Distribution of bigram stems with more than 4 mentions by respondent group**

The bigrams prepared for the previous counts are displayed by stakeholder group. It can be seen that the most common pairs of words occur with similar frequencies across the groups. Note that these are simple counts and are not normalised to the number of respondents in each group. Thus frequency counts are lowest in the clinician (n=3) and regulator (n=2) groups.


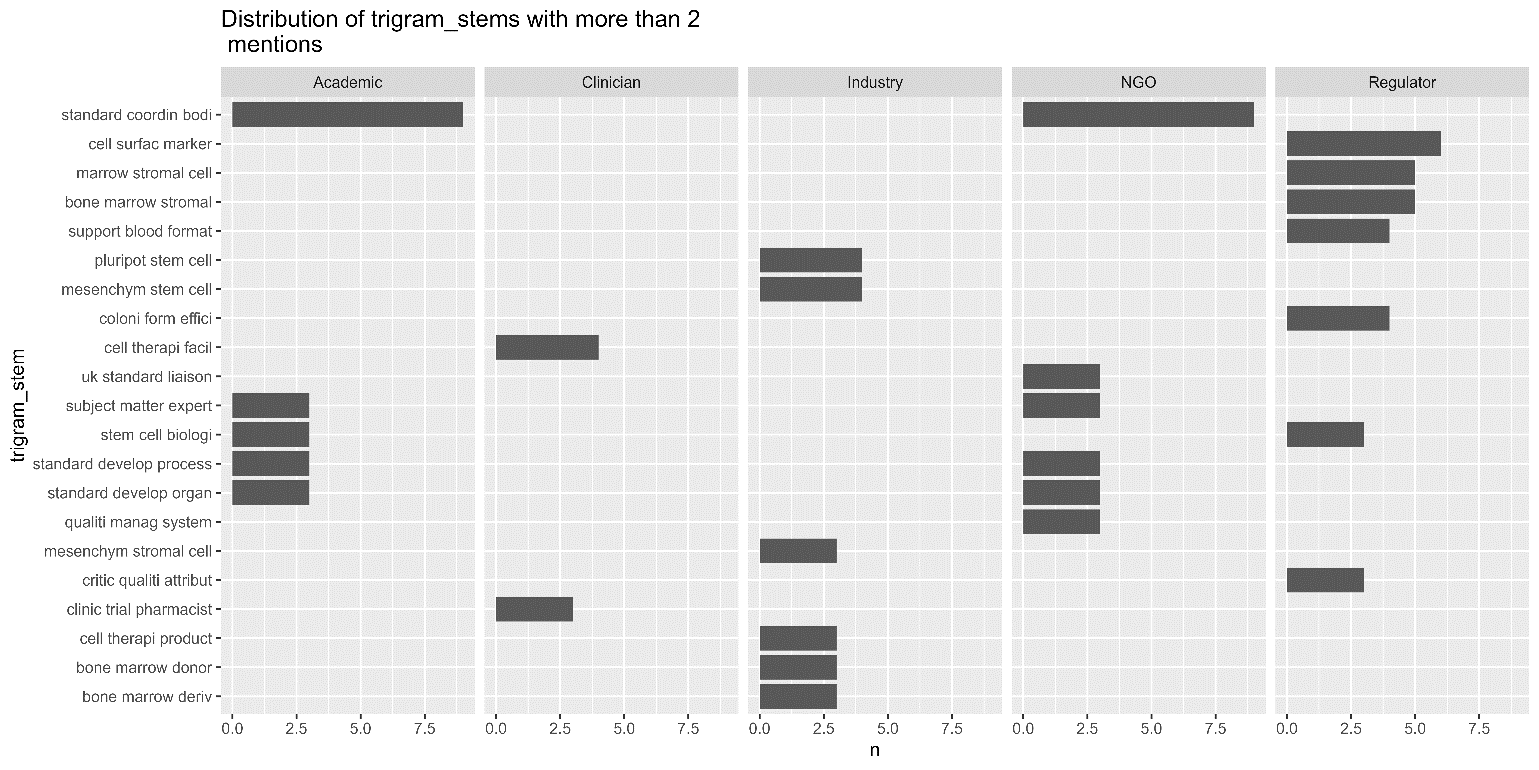


**Figure S9: Distribution of trigram stems with more than 2 mentions by respondent group**

The trigrams prepared for the previous counts are displayed by stakeholder group. Note that these are simple counts and are not normalised to the number of respondents in each group. Thus frequency counts are lowest in the clinician (n=3) and regulator (n=2) groups.
