# Supplementary material for: Attitudes Towards Standardization of Mesenchymal Stromal Cells—A Qualitative Exploration of Expert Views
Source: Stem Cells Transl Med. 2023 Sep 15;12(11):745–57. doi: 10.1093/stcltm/szad056 (PMC10630078; doi:10.1093/stcltm/szad056)
Supplement: szad056_suppl_Supplementary_Information_S2 [file szad056_suppl_supplementary_information_s2.docx]

**Attitudes towards standardization of MSCs – a qualitative exploration of expert views**

Wilson A J, Brown N, Rand E, Genever P G

**SUPPLEMENTARY INFORMATION FILE #2**

1 Participant Information Sheet

2 Informed Consent Form
